# Editorial: Autoinflammatory Diseases: From Genes to Bedside

**DOI:** 10.3389/fimmu.2020.01177

**Published:** 2020-06-19

**Authors:** Ivona Aksentijevich, Alessandra Soriano, José Hernández-Rodríguez

**Affiliations:** ^1^Inflammatory Disease Section, National Human Genome Research Institute, Bethesda, MD, United States; ^2^Division of Internal Medicine, Department of Internal Medicine and Medical Specialties, Azienda Unitá Sanitaria Locale di Reggio Emilia, Arcispedale S. Maria Nuova - IRCCS, Reggio Emilia, Italy; ^3^Clinical Unit of Autoinflammatory Diseases and Vasculitis Research Unit, Department of Autoimmune Diseases, Hospital Clinic, IDIBAPS, University of Barcelona, Barcelona, Spain

**Keywords:** autoinflammatory diseases, genotype/phenotype correlations, inflammasome, ubiquitinopathies, hyperinflammatory state, epigenetics, dermatopathology, treatment

The year 2019 marked the 20th anniversary of the formal recognition of autoinflammatory diseases as a distinct group of rheumatological conditions, following the identification of the gene mutated in patients with a dominantly inherited periodic fever known as familial Hibernian fever (FHF) (1). This nosological concept was introduced by one of the founders of the field, Dr. Daniel Kastner. Prior to this time, the only recognized periodic fever disease was familial Mediterranean fever (FMF) and patients presenting with similar symptoms, irrespective of inheritance pattern, were suspected to have a variant FMF. Most patients, with exception for FMF, were treated with NSAID, glucocorticoids alone, or in a combination with immunosuppressive agents. These chronic life-long conditions negatively impacted patients' quality of life and were associated with significant morbidity and mortality, partially due to treatment-related side effects.

The early advances in the field of autoinflammation were driven by the ascertainment of families with inflammatory phenotypes segregating either as a recessive (FMF) or dominantly (FHF) inherited trait. This allowed for linkage mapping, positional cloning and candidate gene screening even before the completion of human genome sequencing project in 2003. These gene-hunting projects were laborious and time-consuming, but nonetheless successful and led to identification of the first three genes associated with autoinflammatory diseases: *MEFV, TNFRSF1A*, and *CIAS1/NLRP3*.

Familial Mediterranean fever was the first disease to be characterized at the molecular level. FMF, being a common illness in multiple Mediterranean populations, was initially noted in the literature by Galen in the second century AD. Although there were reports of cases with cyclic fevers and pains in the nineteenth century, it remained a mysterious disease and its pathogenesis was attributed to the phases of the moon and other environmental factors. Genetic etiology was not suspected until 1958 when Dr. Harry Heller emphasized the genetic nature of the disease and coined its modern name familial Mediterranean fever (2). The first accurate clinical description of FMF was published in 1945 by Dr. Sheppard Siegal, who reported 10 patients suffering from recurrent bouts of abdominal pain and fevers but who between attacks “may enjoy good health” (3). He named the disease “benign paroxysmal peritonitis.” The next major break-through was in the early nineties, when Drs. Daniel Kastner and Isabelle Touitou undertook finding the causal gene for FMF (4, 5). Independently, they collected many families with FMF to launch genome-wide linkage mapping. This was incredibly ambitious considering available techniques, Southern blot analysis of DNA by restriction fragment length polymorphism (RLFP) markers not dense enough to cover the entire human genome. Complete sequences of many expressed genes were not available. After 7 years of tedious labor, a poorly characterized transcript harboring bi-allelic missense pathogenic mutations associated with FMF was identified. This discovery was celebrated at the First International Meeting on FMF in the summer of 1997 at Jerusalem, which was organized by Dr. Mordechai Pras.

It was not until late nineties that the second periodic fever disease came to attention. Although a five-generation family of the European ancestry with dominantly inherited fevers was described in 1957 by Drs. Bouroncle and Doan (6), most physicians were not aware of the disease. In 1998, the molecular basis of familial Hibernian fever was elucidated with the identification of heterozygous mutations in the *TNFRSF1A* gene (1). The disease was renamed tumor necrosis factor (TNF) receptor-1 associated periodic syndrome (TRAPS) and the term autoinflammation was coined to describe new diseases of the innate immune system (1).

A new era of medical and genetic research in autoinflammation had begun. In 2002, Drs. Hoffman and Kolodner identified heterozygous mutations in the *CIAS1* gene in patients with dominantly inherited cold urticaria (FCAS) and Muckle-Wells (MWS) syndromes, both diseases considered cryopyrin-associated periodic syndromes (CAPS) (7). Another remarkable discovery from this study was that pyrin (encoded by *MEFV*) and the NLRP3 protein share the same N-terminal ~92aa domain denoted as pyrin domain (PYD). This finding suggested the existence of common pathways in the pathogenesis of autoinflammation. Subsequently, a whole family of proteins with the pyrin domain was identified by *in silico* analysis. Many of these proteins function as intracellular sensing receptors to recognize foreign or self-generated danger-associated molecular patterns. They form a molecular complex known as inflammasome that was discovered and characterized in 2002 by the team of late Dr. Jurg Tschopp (8). The initial observation that gain-of-function mutations in the NLRP3 inflammasome lead to increased production of IL-1β formed the basis for genomically-informed therapies. Over time, numerous studies in human cells and murine models showed that IL-1β plays a major role in the pathogenesis of autoinflammatory diseases. These three discoveries established the basis for an entirely new field of investigation.

The next chapter began around 10 years ago with the development of new genomic technologies, next-gene sequencing (NGS), bioinformatics, and the completion of human genome project. These strategies provided researchers and clinicians a variety of tools for gene-hunting projects. Genetic discoveries are now often made in a matter of months if not weeks. The list of genes associated with monogenic autoinflammatory diseases has grown rapidly and currently includes more than 30 genes. Advanced sequencing technologies revealed unusual inheritance patterns including somatic mutations as the cause of adult-onset diseases and cases with digenic inheritance. Digenic inheritance refers to presence of pathogenic mutations in two interacting proteins as the cause of a disease. High-throughput sequencing has brought to light a number of variants with uncertain clinical significance (VUS). Attempts to clarify the clinical implication of these low frequency (1–5%) variants have been carried out through international collaborations. Despite of major accomplishments in dissecting the genetic basis of autoinflammatory conditions, the genetic cause of disease for many patients remains unknown. To complicate things, many of these patients are sporadic cases and it may require orchestrated efforts between multiple research groups to find causal genes.

The field of molecular diagnostics for autoinflammatory diseases has seen substantial growth, providing physicians with the specific information necessary to diagnose and treat patients. There are close to 100 academic and commercial laboratories across the world that perform genetic by testing through single-gene or targeted gene panel analysis, or whole exome sequencing. An international group of experts has been convened in effort to standardize genetic reports and develop consensus guidelines for interpretation of genetic variants.

We have learned a great deal about a wide spectrum of clinical manifestations in patients with autoinflammation. It is likely, as is the case with most human traits, that these phenotypic differences will be explained by modifying gene alleles, epigenetics effects, and environmental factors. Deficiency of adenosine deaminase 2 (DADA2) is case in point: patients may present with fevers, rash, ischemic strokes or with a sole manifestation of pure red cell aplasia. Mutations in the same protein—often in different domains—may give rise to distinct clinical features, therefore these phenotypes need to be referred to in the context of mutant protein e.g., Pyrin-, NOD2-, NLRP3-, or NLRC4-associated diseases. In contrast, a distinct phenotype, such is the case with cold-induced urticaria, could be caused by pathogenic mutations in different genes (*NLRP3, NLRC4, NLRP12, PLCG2, FXII*). About a decade ago an immunological continuum was proposed to designate patients who present with features of autoinflammation and autoimmunity (9). Recent studies described patients with cell-specific features of autoinflammation and/or immunodeficiency.

Studies of molecular pathways in murine models have shed light on broad aspects of the biology of inflammatory responses. Mice deficient for inflammasome components have become instrumental in dissecting signaling pathways that regulate innate immune responses. Studies utilizing pyrin knock-out mice, showed that the pyrin inflammasome has evolved as an innate immune sensor to detect bacterial-induced modifications, which is the first known example of the “guard mechanism” in mammalian innate immunity (10).

A number of “biologicals” have been developed to treat acute inflammation in patients with a broad spectrum of systemic inflammatory diseases. Targeted cytokine therapies, in particular anti-IL1 and anti-TNF, have been efficacious and with minimal side effects in treating patients with autoinflammation even without a known molecular cause of disease. Non-biological drugs such as JAK-STAT inhibitors have recently emerged and are promising in treating patients with interferon-mediated disorders including CANDLE (Chronic Atypical Neutrophilic Dermatosis with Lipodystrophy and Elevated Temperature) syndrome, STING-associated vasculopathy with onset in infancy (SAVI) and Aicardi-Goutieres syndrome (AGS).

The term autoinflammation has spread beyond the boundaries of internal medicine and rheumatology, and is now used in disciplines of dermatology, immunology, and neurology. Dysregulation of the innate immune system is increasingly considered to have role in the pathogenesis of many human conditions, including common multifactorial cardiovascular, metabolic, neurodegenerative, autoimmune diseases, and in particular in polygenic or systemic inflammatory diseases, such as Behçet disease and periodic fever, aphthous stomatitis, pharyngitis and cervical adenitis (PFAPA) syndrome. A better understanding of the molecular mechanisms underlying dysregulation of the innate immune system will provide a foundation for developing more affordable and effective treatments. Witnessing these developments has been incredibly rewarding for those of us in the field and it will be exciting to see where it goes from here.

In the present issue of Frontiers in Immunology, “Autoinflammatory diseases: from genes to bedside,” the first monographic issue about autoinflammatory diseases, several investigators have contributed with original and review articles covering genetic, pathogenic, epigenetic, clinical and therapeutic aspects of different autoinflammatory conditions.

The “genes” part of the topic explores relevant genetic, pathogenic and epigenetic mechanisms implicated in autoinflammatory diseases. Martorana et al.. review the most common mutations and the evidences of genotype/phenotype correlations of the main monogenic autoinflammatory diseases. The role of NLRP3 and pyrin inflammasomes in the pathogenesis of CAPS, and FMF and pyrin-associated autoinflammation with neutrophilic dermatosis (PAAND), respectively, has been addressed by de Torre-Minguela et al.. Aksentijevich and Zhou describe the latest advances on the pathogenic mechanisms of ubiquitinopathies, a new category of autoinflammatory diseases involved in the NF-κB pathway, which include linear ubiquitin chain assembly complex (LUBAC) and OTULIN deficiencies, and haploinsufficiency of A20. Carta et al.. propose two different pathways of inducing abnormal IL-1β production in autoinflammatory diseases depending on the cell type affected, in which the authors postulate that professional inflammatory cells would cause a direct inflammatory response and non-immune cells may participate indirectly in the inflammatory cascade by releasing stress signals that trigger and propagate inflammation. In the same sense, Gül reviews the concept of autoinflammation and uses it for monogenic and polygenic autoinflammatory diseases associated with seemingly unprovoked inflammatory episodes mediated mainly by the innate immune system. In addition, Gül also proposes and expands nomenclature by using the concept of “hyperinflammatory” state for those disorders characterized by episodes of exaggerated inflammatory response only when triggered by certain factors or situations. Álvarez-Errico et al.. review the recent advances on the contribution of epigenetic mechanisms in the disease expression of some autoinflammatory diseases.

The “bedside” part of the topic reviews important clinical and translational research, and therapeutic contributions in autoinflammatory diseases. Özen et al.. wrote a comprehensive overview about what are still considered unsolved problems in FMF, such as the involved mechanism of the disease, inheritance patterns and treatment in colchicine resistant patients. Ruiz-Ortiz et al.. make an original contribution in characterizing clinical manifestations associated with the low-penetrance R92Q variant in *TNFRSF1A* and differentiating disease phenotypes between patients with pediatric and adult onset. In an article about CANDLE syndrome, Torrelo et al. reviews in depth all the pathophysiological, clinical, and biologic features of this complex monogenic interferonopathy. The cytokine signature in patients with Behçet disease is explored by Lopalco et al., who suggest an increased signature of IL-6, TNF-α, and Th17 in patients with mucocutaneous and uveitis manifestations. Cantarini et al.. propose a set of clinical diagnostic criteria for adult-onset PFAPA syndrome with a high-predictive potential for identifying PFAPA patients among subjects with fever of unknown origin. Finally, in two review articles, Figueras-Nart et al.. analyze the most remarkable dermatologic and dermatopathologic features of monogenic autoinflammatory diseases by using a classification based on the predominant cutaneous lesion, and Soriano et al. describe the current treatment of the most frequent monogenic autoinflammatory diseases and PFAPA syndrome based on the best available evidence and also propose a practical guide to their management.

This first monography entirely dedicated to autoinflammatory diseases provides stimulating information on many of the past, present and future advances and challenges in this field.

## Author Contributions

All authors have made a substantial, direct and intellectual contribution to the work, and approved it for publication.

## Conflict of Interest

The authors declare that the research was conducted in the absence of any commercial or financial relationships that could be construed as a potential conflict of interest.
